# Identification of MicroRNAs in *Taxillus chinensis* (DC.) Danser Seeds under Cold Stress

**DOI:** 10.1155/2021/5585884

**Published:** 2021-05-30

**Authors:** Jine Fu, Lingyun Wan, Lisha Song, Lili He, Ni Jiang, Hairong Long, Juan Huo, Xiaowen Ji, Ying Wei, Shugen Wei, Limei Pan

**Affiliations:** Guangxi Botanical Garden of Medicinal Plants, Nanning, China 530023

## Abstract

*Taxillus chinensis* (DC.) Danser, a parasitic plant that belongs to the Loranthaceae family, has a long history of being used in the Chinese medicine. We observed that the loranthus seeds were sensitive to temperature and could lose viability below 0°C quickly. Thus, we performed small RNA sequencing to study the microRNA (miRNA) regulation in the loranthus seeds under cold stress. In total, we identified 600 miRNAs, for the first time, in the loranthus seeds under cold stress. Then, we detected 224, 229, and 223 miRNAs (TPM > 1) in A0 (control), A1 (cold treatment for 12 h at 0°C), and A2 (cold treatment for 36 h at 0°C), respectively. We next identified 103 differentially expressed miRNAs (DEmiRs) in the loranthus seeds in response to cold. Notably, miR408 was upregulated during the cold treatment, which can regulate genes encoding phytocyanin family proteins and phytophenol oxidases. Some DEmiRs were specific to A1 and may function in early response to cold, such as gma-miR393b-3p, miR946, ath-miR779.2-3p, miR398, and miR9662. It is interesting that ICE3, IAA13, and multiple transcription factors (e.g., WRKY and CRF4 and TCP4) regulated by the DEmiRs have been reported to respond cold in other plants. We further identified 4, 3, and 4 DEmiRs involved in the pathways “responding to cold,” “responding to abiotic stimulus,” and “seed development/germination,” respectively. qRT-PCR was used to confirm the expression changes of DEmiRs and their targets in the loranthus seeds during the cold treatment. This is the first time to study cold-responsive miRNAs in loranthus, and our findings provide a valuable resource for future studies.

## 1. Introduction

Loranthus (*Taxillus chinensis* (DC.) Danser) belongs to the *Loranthaceae* family and is a parasitic plant that grows by attacking other plants like *Aceraceae*, *Anacardiaceae*, *Euphorbiaceae*, *Fabaceae*, *Fagaceae*, *Juglandaceae*, *Moraceae*, *Rosaceae*, and *Rutaceae* [1]. It is mainly distributed in China southern and southwestern areas and is also named as “Sang Ji Sheng” in Chinese. Loranthus has a long history of being used in the Chinese medicine as their leaves and stems can be used for the treatments of rheumatism, threatened abortion, hypertension, angina pectoris, stroke, and arrhythmia [1]. However, our knowledge about this plant is very limited. It is important to study loranthus in terms of their growing conditions, development, and responses to biotic stresses.

MicroRNAs (miRNA) are a class of endogenous small noncoding RNAs (18~24 nt) that regulate protein expression on the posttranscriptional level *via* messenger RNA (mRNA) cleavage or translational repression [2, 3]. In plants, miRNA primary transcripts are stabilized by DAWDLE and processed to the miRNA : miRNA^∗^ duplexes by DCL1, HYL1, SE, and nuclear CBC in D-bodies [4]. The miRNA : miRNA^∗^ duplexes are next methylated by HEN1 and transported to the cytoplasm [5]. One strand of the duplex, as the mature miRNA, is incorporated into an AGO protein complex [4]. Until now, the miRBase (v22.1) has documented 10,414 miRNA and miRNA^∗^ sequences for 82 plant species, such as rice, maize, Arabidopsis, and grape. However, probably due to the inaccessible of the loranthus genome, our knowledge about the miRNA sequences and their regulation in the loranthus is still blank.

Among the abiotic stresses, cold stress, including chilling (<20°C) and freezing (<0°C), is an important factor that affects the geographical distribution, plant season, and production of many plants, especially for tropical and subtropical crops [6]. Studies have reported the miRNAs associated with the response to cold stress in some plants. For example, miR168, miR397, miR164, and miR1029 were differentially expressed in wheat genotype C-306 under cold stress [7]. In Arabidopsis, Tiwari et al. identified 93 cold-responsive differentially expressed miRNAs (e.g., miR160, miR169, miR408, and miR779.1) and identified that the target genes of these miRNAs encoded proteins acting in the transcriptional regulation [3]. To understand the miRNA functions under cold stress in *Populus tomentosa*, Chen et al. compared the miRNA expression profiles of plantlets treated or not with cold conditions (4°C for 8 h) and identified 30 differentially regulated miRNAs from 11 miRNA families, including miR167, miR395, and miR1450 [8]. Using microarray technology, Lv et al. identified 18 cold-responsive rice miRNAs, including miR156k, miR166k, miR166m, miR167a/b/c, miR168b, miR169e, miR169f, miR169h, miR171a, miR535, miR319a/b, miR1884b, miR444a.1, miR1850, miR1868, miR1320, miR1435, and miR1876 [9]. Yang et al. identified 62 miRNAs, such as miR1310, miR157a, miR5077, miR169, miR397-5p, and miR863-5p, with different expression in sugarcane response to low-temperature environment [10]. However, we know little about the miRNA regulation mechanisms in loranthus under cold stress.

Small RNA sequencing has been widely used to identify known and novel miRNAs in model and nonmodel plants, like *Abelmoschus esculentus* [11]. Previously, we reported the transcriptome of loranthus seeds in response to water loss [12] and this transcriptome can be used for miRNA discovery in loranthus, like rice [13] and sugarcane [14]. In this study, we first evaluated the viabilities of loranthus seeds kept under various temperature range for different time lengths. Then, small RNA sequencing was performed to identify known and novel miRNAs in the loranthus seeds. Comparison of miRNA profiles identified dysregulated miRNAs involved in the response to cold stress. This is the first time to study miRNAs in loranthus, and our study will provide a valuable resource for future studies. The output of this study will improve our understanding towards the miRNA regulation mechanisms under cold stress in loranthus seeds.

## 2. Materials and Methods

### 2.1. Seed Collection

The seeds of *Taxillus chinensis* (DC.) Danser were collected from ten trees of mulberry *Morus alba* in the experimental filed of Guangxi Botanical Garden of Medicinal Plants (Guangxi, China) and were confirmed by a senior botanist. The seeds were deposited in the herbarium of Guangxi Botanical Garden of Medicinal Plants (accession number: s0001794). Seeds with similar appearance (no diseases, insect pests and mechanical damage, maturity, and plumpness) were selected, peeled, and washed with sterile water. No specific permits were required for the described field studies. The location is not privately owned or protected in any way, and the field studies did not involve endangered or protected species.

### 2.2. Determination of Seed Viability by Staining

First, for each following cold treatment, we selected 100 loranthus seeds and treated them with different temperature conditions (-20°C, -5°C, -1°C, 0°C, 4°C, 10°C, and 25°C) for different time lengths (1 d, 2 d, 3 d, 4 d, 5 d, 10 d, and 20 d). Then, they were taken out for the viability test by immersing the seeds in a solution of 1% (w/v) 2,3,5-triphenyl tetrazolium chloride (TTC, Sigma), as previously described [12]. Briefly, 100 seeds were cut by a sterile scalpel for small incisions that allow the TCC to enter. After the seeds were incubated in the 1% TCC solution for 8 h at 25°C, they were washed several times by sterile water. If viable, a redox reaction would change the embryo color from white to reddish brown during cellular respiration. Then, another cold treatment experiment was conducted at a temperature range (-4°C to 25°C) for a short time (1 h, 2 h, 3 h, 4 h, 5 h, 10 h, 12 h, 16 h, 20 h, 24 h, 30 h, 36 h, 40 h, and 48 h), and the viability of the cold treated seeds was tested.

### 2.3. RNA Isolation, Library Construction, and Small RNA Sequencing

TRIzol reagent (Invitrogen) was used to extract total RNA from the seeds (100 mg, in triplicates) stored under 0°C for 0 h (A0), 12 h (A1), and 36 h (A2), as previously described [12, 15]. The quantity and quality of total RNA were evaluated by the Agilent Bioanalyzer 2100 (Agilent Technologies). Then, total RNA (1 *μ*g) of each sample was used to construct the small RNA libraries using the MGIEasy Small RNA Library Prep Kit (cat # 1000005269), as previously described [16]. Briefly, total RNA was initially fractionated on a 15% urea-PAGE gel electrophoresis, and a band corresponding to small RNAs (18–30 bp) was excised. After small RNAs were extracted by centrifugation, they were ligated with the adenylated 3′ adapter. Then, RT primer with barcode was used to anneal the 3′ adenylated adapter, followed by the ligation of 5′ adapter and the reverse transcript reaction. After first strand cDNA synthesis, we amplified the product by 15 cycles and carried out another size selection (103–115 bp) from the gel. After the gel purification, the PCR product was quantified by Qubit (Invitrogen) using the Qubit dsDNA HS Assay Kit and denaturized. Then, the product was mixed with the Single Strand Circularization Reaction Mixture (11.6 *μ*L Splint Buffer and 0.5 *μ*L DNA Rapid Ligase), vortexed 3 times (3 s each), and centrifuge briefly to collect the solution to the bottom of the tube. After an enzymatic digestion and a cleanup of the enzymatic digestion product, the final product (single strand circular DNA) was quantitated with Qubit ssDNA Assay Kit. For small RNA sequencing, we first generated the DNA nanoballs (DNBs) with ssDNA circles by rolling circle replication (RCR) to enlarge the fluorescent signals at the sequencing process, as previously described [17]. Then, the DNBs were loaded into the patterned nanoarrays, and single-end read of 50 bp was read through on the BGISEQ-500 platform.

### 2.4. Data Cleaning, miRNA Identification, Expression Profiling, and Differential Expression Analysis

The raw reads were cleaned by SOAPnuke, as previously described [18]. Then, clean data of all the samples were merged into one file, and reads less than 30 counts were filtered. Next, we used miRDeep2 to predict the potential miRNA hairpins in the previously published loranthus seed transcriptome [12, 19]. Then, predicted miRNA precursors were used as reference to profile the miRNA expression, as previously described [16]. To identify more loranthus miRNAs, we next compared all the clean data with the mature plant miRNAs in miRBase (v22.1) and maximal 2 mismatches were allowed. Read with highest expression aligned to each miRNA family was selected as the miRNA sequence (A) for loranthus. Then, the reads aligned to the miRNA sequence (A) were counted as the expression for this family, and TPM (transcripts per million reads) method was used for normalization. Differential expression was performed using edgeR with following cut-offs [16]: TPM > 1, log 2 fold change (log2FC) > 1 or < -1, *p* value <0.05, and FDR (false discovery rate) < 0.01.

### 2.5. miRNA Target Prediction and Functional Analysis

Previously published loranthus seed transcriptome was used as the resource for miRNA target genes, and three software (psRobot, TarHunterL, and TargetFinder) were used to predict the target genes of differentially expressed genes with default parameters [20, 21]. Targets identified by more than two software were considered as final targets for a miRNA. Then, we applied the Gene Ontology and KEGG pathway annotation for the seed transcriptome to annotate the target genes. Enriched GO terms and pathways were identified using two statistical values—*p* value (calculated by Fisher's exact test, <0.05) and *q* value (calculated by the R package “*q*value,” <0.05), as previously described [12].

### 2.6. qRT-PCR

We performed quantitative real-time PCR (qRT-PCR) to validate the expression levels of candidate miRNAs and their targets using the poly-A tail extension method. A total of 6 miRNAs (miR156r, aly-miR170a-3p, miR393, miR408, miR1520o-3p, and miR8036) and two targets (ICE3 and TCP4 TF) were selected. The primers for these candidates were synthesized at BGI-Shenzhen (Shenzhen, China). The miRNA First Strand cDNA Synthesis (Tailing Reaction) reagent (B532451, Sangon Biotech, Shanghai, China) was used to add poly-A tail to the RNA and to amplify the total RNA to cDNA product. Diluted cDNA product (10 times) was then used as template for the qRT-PCR experiment with the MicroRNA qPCR Kit (SYBR Green, B532461, Sangon, Shanghai, China), following the manufacturer's protocol. The qRT-PCR reactions were conducted, and the signals were read on the qTOWERE2.2 PCR machine (AnalytikJena, Germany). We sued ∆Ct and ∆∆Ct values to show the expression level of a miRNA/mRNA in one sample and the relative normalized expression (RNE, RNE = 2^−∆∆Ct^) of a miRNA/mRNA in two samples, respectively, as previously described [16].

## 3. Results

### 3.1. Seed Collection, Cold Treatment, and Germination Experiments

**Figure 1:**
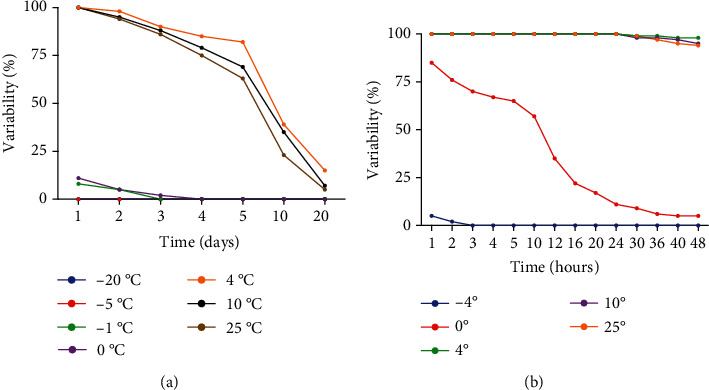
Cold treatment of the loranthus seeds. (a) Loranthus seeds under larger range of temperature (-20°C to 25°C) of cold treatment (1 d to 20 d). (b) Loranthus seeds kept under various temperature range (-4°C to 25°C) for short period (1 h to 48 h).

To identify miRNAs in loranthus seeds and to study the association of miRNAs with the germination capacity of loranthus seeds under cold stress, we obtained loranthus seeds from the experimental filed of Guangxi Botanical Garden of Medicinal Plants (Guangxi, China). Initially, we stored the seeds under multiple temperature conditions (-20°C, -5°C, -1°C, 0°C, 4°C, 10°C, and 25°C) for different time lengths (1 d, 2 d, 3 d, 4 d, 5 d, 10 d, and 20 d). Interestingly, we observed that the loranthus seeds are sensitive to cold stress and that very few seeds can survive; then, the temperature was below -1°C (Figure 1(a)). Also, the viability test showed that the seeds can be stored above 4°C for some days (Figure 1(a)), and the viability decreased probably due to the loss of water. Based on these data, we chose a temperature range (-4°C to 25°C) for the following experiments and stored the seeds for a short time (1 h, 2 h, 3 h, 4 h, 5 h, 10 h, 12 h, 16 h, 20 h, 24 h, 30 h, 36 h, 40 h, and 48 h). The viability test of loranthus seeds in this experiment (Figure 1(b)) showed that -4°C was not be suitable for loranthus seed storage and that 0°C could be a good point to study the loranthus under cold stress. Then, we extracted the total RNA from the seeds stored under 0°C for 0 h (A0), 12 h (A1), and 36 h (A2) and sent them for small RNA sequencing.

### 3.2. miRNA Identification and Expression Profiles

**Figure 2:**
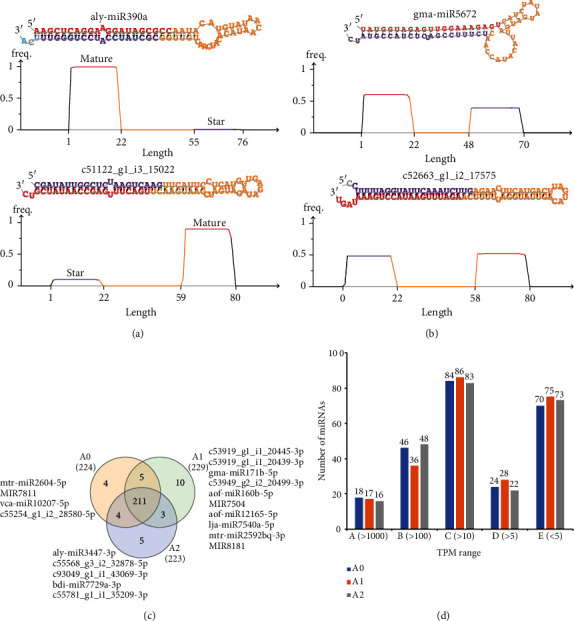
miRNAs identified in the loranthus seeds under cold stress. (a) Conventional structure of loranthus miRNAs identified by miRDeep2. (b) Possible siRNA-like miRNAs identified for the loranthus seeds. (c) Venn diagram of miRNAs identified in the loranthus seeds under 0°C for 0 h (A0), 12 h (A1), and 36 h (A2). (d) Distribution of miRNA expression identified for each sample.

After data cleaning, we obtained a total of 327.8 million reads for all samples (average 36.42 million reads). After the clean data of all samples were merged in to one file, it was used to identify miRNAs in the loranthus seeds using miRDeep2 [19]. Initially, we obtained 219 unique miRNA precursors which can produce 442 mature/passenger miRNAs (Supplementary Table S1). Homolog miRNA names or the transcript names were used as the names of loranthus miRNAs. From the miRDeep2 result, we found that the loranthus miRNAs have conventional structures. For example, aly-miR390a and c51122_g1_i3_15022 not only have the hairpin structure but also have higher expression of the mature miRNAs than the passenger miRNAs (Figure 2(a)). Interestingly, from the seed transcriptome, we also identified some siRNA-like structures (Figure 2(b)). The expression levels of small RNAs produced from both arms of the siRNA-like structures were similar, such as gma-miR5672 and c52663_g1_i2_17575. To further identify loranthus miRNAs, we compared the clean data to all the plant mature miRNAs in miRBase (v22.1) and obtained 158 miRNAs from different families (Supplementary Table S1). Thus, in total, we identified 600 miRNAs, for the first time, in the loranthus seeds under cold stress (Supplementary Table S1).

We next profiled the miRNA expression in the loranthus seeds under cold stress. After lowly expressed miRNAs (average TPM < 1) were filtered, we identified a total of 242 miRNAs in the loranthus seeds, of which 224, 229, and 223 distributed to A0, A1, and A2, respectively (Figure 2(c), Supplementary Table S2). It is not surprised that a large proportion of miRNAs were shared and that we found 211 in all three samples (Figure 2(c)). Notably, we found 4 (e.g., mtr-miR2604-5p), 10 (e.g., miR7504 and miR8181), and 5 (e.g., bdi-miR7729a-3p) miRNAs specifically identified in A0, A1, and A2, respectively (Figure 2(c)). Then, we analyzed the expression levels of miRNAs for each sample, and Figure 2(d) showed that 18, 17, and 16 miRNAs were expressed more than 1000 TPM in A0, A1, and A2, respectively.

### 3.3. Cold-Responsive miRNAs in Loranthus Seeds

**Figure 3:**
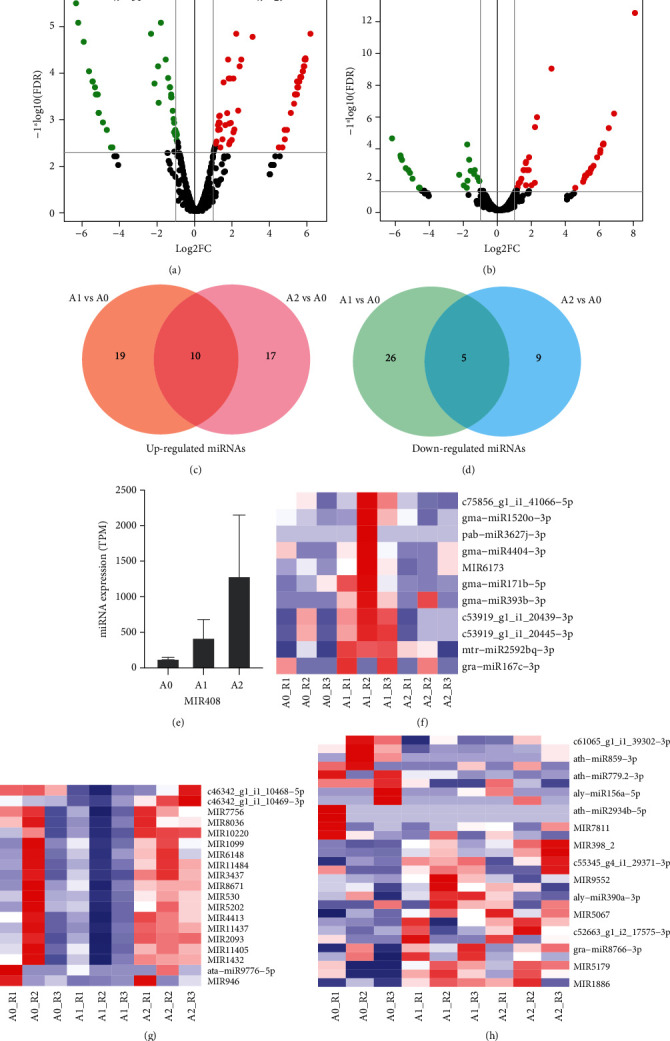
Differentially expressed miRNAs identified in the loranthus seed under cold stress. (a) Volcano plot of DEmiRs identified in A1 compared to A0. (b) Volcano plot of DEmiRs identified in A2 compared to A0. (c) Comparison of upregulated miRNAs identified in A1 and A2 compared to A0. (d) Comparison of downregulated miRNAs identified in A1 and A2 compared to A0. (e) Expression levels of miR408 in the loranthus seeds under cold treatment. (f) Lowly expressed miRNAs identified in A1 but highly expressed in A0 and A2. (h) A1 specifically identified DEmiRs might be involved in the early response to cold of the loranthus seeds.

We next analyzed the differentially expressed miRNAs (DEmiRs) in the loranthus seeds under cold stress. Compared to A0, we identified 60 miRNAs differentially expressed in A1 (29 upregulated and 31 downregulated) (Figure 3(a)), including miR946, ath-miR779.2-3p, miR398, miR9662, and miR408 (Supplementary Table S3). Then, we identified 41 differentially expressed miRNAs (27 upregulated and 14 downregulated) in A2 compared to A0 (Figure 3(b)). Comparison of DEmiRs in A1 and A2 showed that 10 upregulated (Figure 3(c), Table 1) and 5 downregulated (Figure 3(d), Table 1) miRNAs shared. The most upregulated and downregulated miRNAs were miR1886 and miR2865, respectively.There were 19 miRNAs specifically upregulated in A1, such as aly-miR390a-3p, aof-miR160b-5p, gma-miR171b-5p, and gra-miR167c-3p (Supplementary Table S3), which might be involved in the early response to cold, while the 17 miRNAs specifically upregulated in A2 include aly-miR3447-3p, ath-miR3434-5p, miR1508, miR5813, and sly-miR10539-3p (Supplementary Table S3). Likewise, we identified 26 (e.g., aly-miR156a-5p and miR530) and 9 (e.g., miR157, miR169_2, and miR918) miRNAs specifically downregulated in A1 and A2, respectively (Supplementary Table S3).

Then, we compared the miRNA expression profiles of A1 and A2. edgeR identified 43 upregulated and 23 downregulated miRNAs in A2 compared to A1 (Supplementary Table S3), which might be related to apoptosis. Notably, miR408 was found to be upregulated in A2 compared to A1 (Table 1, Figure 3(e)). Interestingly, we identified 11 miRNAs peaked at 12 h in the loranthus seeds during the cold treatment (Figure 3(f), Supplementary Table S3), such as gma-miR171b-5p, c53919_g1_i1_20445-3p, and gma-miR393b-3p. Also, the expression of 19 miRNAs (e.g., ata-miR9776-5p, c46342_g1_i1_10468-5p, miR530, and miR946) reached the low point at 12 h during the cold treatment (Figure 3(g), Supplementary Table S3). Further, we identified 29 miRNAs differentially expressed in A1 compared to A0 but not changed between A2 and A1, including 10 upregulated miRNAs and 19 downregulated miRNAs (Figure 3(h), Supplementary Table S3).

### 3.4. Target Prediction and Pathway Analysis

We next analyzed the potential target genes for all the loranthus miRNAs using psRobot, TargetFinder, and TarHunterL. Initially, 8,377, 3,441, and 10,144 target genes were predicted to be regulated by 502, 369, and 519 miRNAs using TarHunterL, TargetFinder, and psRobot, respectively. Then, we filtered the miRNA-target pairs only identified by one software and obtained 5,610 targets for 479 miRNAs (Table 2). Using this dataset, we identified 841 targets for 90 DEmiRs (Supplementary Table S4). It showed that 501 target genes were regulated by 53 DEmiRs in A1 vs. A0, including 316 target genes for 24 upregulated miRNAs and 185 target genes for 29 downregulated miRNAs (Table 2). These miRNA-mRNA pairs include miR1886~c55724_g4_i3 (HSF domain class transcription factor), miR8036~c43064_g1_i1 (transcription factor TCP4), gma-miR1520o-3p~c43266_g1_i1 (ethylene-responsive transcription factor CRF4), and miR946~c54913_g2_i2 (membrane magnesium transporter). Unlike A1, we only identified 216 target gens for 36 DEmiRs in A2 vs. A0, including 180 targets for 24 upregulated miRNAs and 39 targets for 12 downregulated miRNAs (Table 2). Among them, c50764_g1_i3 (auxin-responsive IAA13-like protein) and c54548_g3_i3 (SEC1 family transport protein) were regulated by miR5067 and ath-miR859-3p, respectively. We also identified 331 and 293 loranthus genes that can be targeted by 37 upregulated and 19 downregulated miRNAs (Table 2), respectively, in A2 vs. A1.

**Figure 4:**
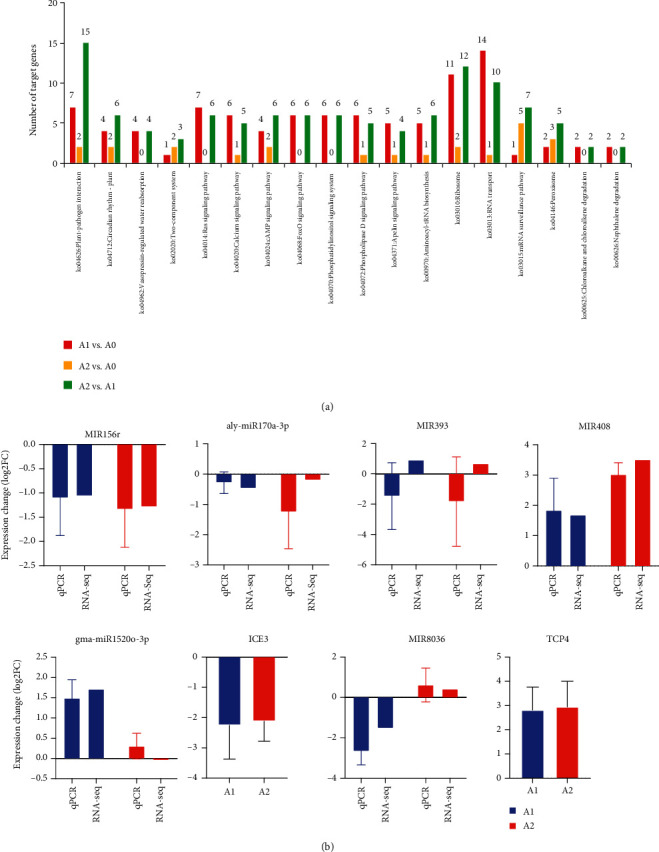
KEGG pathway analysis of DEmiRs and qRT-PCR validation. (a) KEGG pathway enrichment by the target genes of DEmiRs identified in A1 and A2 compared to A0. (b) qRT-PCR experiment for two miRNA~mRNA pairs (gma-miR1520o-3p~ICE3 and miR8036~TCP4) and another four miRNAs (miR156r, aly-miR170a-3p, miR393, and miR408).

Next, we performed the KEGG pathway analysis for the target genes of DEmiRs identified in each comparison. For most of the pathways, the numbers of target genes for DEmiRs in A1 vs. A0 and A2 vs. A1 were similar. For example, “Circadian rhythm-plant” (ko04712), “Phosphatidylinositol signaling system” (ko04070) and “Ribosome” (ko03010) involved similar numbers of targets genes of the DEmiRs identified in A1 vs. A0 and A2 vs. A1 (Figure 4(a)). However, we found some pathways might be regulated by some DEmiRs identified only in A2. For example, 15 target genes of DEmiRs in A2 vs. A1 were involved in the pathway “Plant-pathogen interaction” (ko04626), and this number for A1 vs. A0 was 7 (Figure 4(a)). More interestingly, 5 and 7 target genes related to “mRNA surveillance pathway” (ko03015) were identified for the DEmiRs in A2 vs. A0 and A2 vs. A1, respectively (Figure 4(a)), but we only found 1 target gene from this pathway regulated by the DEmiRs in A1 vs. A0 (Figure 4(a)).

### 3.5. Key miRNAs and Their Regulation Networks

Based on the Gene Ontology annotation for miRNA target genes, we identified 4 DEmiRs (miR4228, miR8036, aly-miR390a-3p, and zam-miR164d-5p) targeting 7 loranthus genes that were involved in the response to cold (Table 3). Among these DEmiRs, aly-miR390a-3p was upregulated in the loranthus in A1 and remained the high expression in A2; the expression of miR4228 and miR8036 was upregulated in A2 compared to A1. Likewise, we identified 3 DEmiRs (gma-miR1520o-3p, c53051_g1_i1_18088-5p, and sly-miR10539-3p) as the regulators of 3 genes involved in the response to abiotic stimulus (Table 3). Interestingly, gma-miR1520o-3p and sly-miR10539-3p were upregulated in A1 vs. A0 but downregulated in A2 vs. A1. These two miRNAs might be key regulators of loranthus seeds to defend the cold stress, and their downregulation might trigger the apoptosis pathways. Further, we analyzed the DEmiRs related to seed development and germination. As shown in Table 3, miRNAs were found to regulate the biological processes of embryo development ending in seed dormancy, seed germination, seed dormancy process, mucilage extrusion from seed coat, and mucilage metabolic process involved in seed coat development. An important finding was that two miRNAs (ath-miR3434-5p and miR5998) regulating the embryo development ending in seed dormancy were upregulated in A2. Another key finding was that miRNAs regulating the seed development and germination processes were upregulated in A2. These indicate that cold treatment for 36 hours can dramatically damage the loranthus seeds viability, which was consistent with the physiology experiment.

### 3.6. qRT-PCR

We performed the qRT-PCR to validate the expression levels of miRNAs and their target genes in the loranthus seeds under cold stress. Two pairs of miRNA~targets (miR1520-3p~ICE1 and miR8036~TCP4 TF) and another four miRNAs (miR156r, aly-miR170aa-3p, miR3434-5p, miR393, and miR408) were selected, and the U6 RNA was used as internal control. To compare the expression changes of miRNAs/genes in the samples, we used log2 values (relative normalized expression and fold change for qRT-PCR and deep sequencing, respectively). Among the miRNA candidates, aly-miR179a-3p and MIR393 were not changed significantly in the loranthus seeds during the cold treatment and their expression levels were confirmed by qRT-PCR (Figure 4(b)). The downregulation of miR156r and the upregulation of miR408 were agreed by both RNA-Seq and qRT-PCR (Figure 4(b)). It is notable that the upregulation of gma-miR1520o-3p and the downregulation of its target (ICE3) were confirmed by qRT-PCR (Figure 4(b)). Likewise, the regulation of miR8036~TCP4 was observed (Figure 4(b)). Overall, the miRNA expression changes were confirmed by qRT-PCR with the ratio 75% (9 out of 12). The high agreement of qRT-PCR and RNA-Seq indicates the miRNAs identified in this study might be functional in response to cold.

## 4. Discussion

Our study is aimed at providing insights into the cold-responsive miRNAs of loranthus seeds. Using the small RNA sequencing data, we first identified miRNAs for loranthus as they are not accessible in the public databases, such as miRBase. In total, we identified 600 miRNA/miRNA^∗^ sequences for the first time in the loranthus seeds (Supplementary Table S1). Among them, the miRDeep2 predicted 442 miRNA/miRNA^∗^ sequences were produced from 219 pre-miRNAs. They showed high similarity with other miRNAs in terms of the sequence and/or the structure (Figures 2(a) and 2(b)), while the rest 158 miRNAs only had sequence similarity with other known miRNAs due to the missing of loranthus genome (Supplementary Table S1). These methods for miRNA discovery have been applied in other studies. For example, Li et al. identified 93 conserved miRNAs and 454 novel miRNAs in sugarcane using their previously reported transcriptome as reference [14]. Zhang et al. found 27 putative pre-miRNAs using the rice transcriptome as reference [13]. Another sugarcane study aligned the small RNA sequencing reads against the known miRNAs in miRBase and identified 209~219 known miRNAs [10]. However, the miRNAs identified in our study still require more experiments to be validated.

Cold stress suppresses the plant growth via the inhibition of metabolic reactions and leads to osmotic, oxidative, and other stresses [22]. It is one of the major factors that limit plant distribution, yield, and quality [6]. The cold signaling pathways have been well studied in plants, such as signaling transduction [6]; however, the regulatory roles of miRNAs in the response to cold are not clear. We identified a total of 103 DEmiRs (Supplementary Table S3) in the loranthus seeds under cold stress. Among them, some miRNAs have been reported to be functional in the cold tolerance of plants. For example, miR393 showed downregulation in wheat under cold [7] but overexpression of miR393 can improve the cold tolerance and tillering of switchgrass via the auxin signaling transduction [23]. In the present study, we observed that compared to A0, miR393 was upregulated in A1 but then its expression went down in A2 (Figure 3(b), Supplementary Table S3). The expression of miR393 might be a marker to evaluate the cold response of loranthus seeds and indicated that after 36 h cold treatment, the seeds might enter the period of dormancy or death. In Arabidopsis, ICE1 (inducer of CBF expression 1) is experimented to be induced by chilling and freezing tolerance [24]. miR397 has been shown to be a positive regulator via the CBF-dependent signaling pathway, and overexpression of miR397a can increase the CBF expression which leads the induction of cold-responsive genes [25]. In the present study, we identified two miR397 members (eun-miR397a and aly-miR397b) but their expression was less than 1 TPM. Interestingly, target prediction showed that another miRNA gma-miR1520o-3p can regulate c25755_g2_i1 (inducer of CBF expression 3) (Supplementary Table S4). Like miR393, the expression of gma-miR1520o-3p peaked in A1. This indicates that our results are consistent with previous studies. However, the functions of DEmiRs in loranthus seeds under cold stress require further experiments to be explored.

miR408 is a highly conserved miRNA in plants and has been recognized to be induced by cold and other abiotic stresses [3]. It has the potential of regulating the genes encoding phytocyanin family proteins (e.g., cupredoxin, plantacyanin, and uclacyanin) [26] and phytophenol oxidases (laccases) [27], which are oxidize flavonoids during seed development and environmental stress [28]. In Arabidopsis, high miR408 expression can improve the cold tolerance and can enhance the cellular antioxidant capacity [29]. We found miR408 increased in the loranthus seeds during the cold treatment (Figure 3(e)). Considering the seed viability of A2 was very limited (Figure 1(b)), miR408 might play a central function in plant survival, like Arabidopsis [29] and *Populus simonii*×*P. nigra* [30]. Target prediction showed that miR408 can regulate the loranthus transcript c9593_g1_i1 (Supplementary Table S4), which was not aligned to other databases.

Transcription factors (TFs) are another large group of proteins involved in the cold response in plants. For example, genes encoding Ap2-like ethylene-responsive transcription factor and nuclear transcription factor Y subunit A-3 regulated by miR172 and miR169, respectively, were differentially expressed between cold-sensitive and cold-resistant tomato genotypes [31]. Interestingly, MYB and TCP TFs were found to express more and earlier in the cold-sensitive sugarcane varieties compared to the cold-tolerant varieties [32]. WRKY TFs were significantly changed in multiple tissues and identified to regulate cold resistance in *Prunus mume* [33]. In the present study, we observed that 4 TF subtypes (e.g., HSF domain class, WRKY, ethylene-responsive, and TCP4) were regulated by 5 miRNAs (Supplementary Table S4), including miR1886, miR2084, miR6135, miR8036, and gma-miR1520o-3p. Compared A0 miR1886 (targeting HSF domain class TFs) was upregulated, and miR8036 (targeting TCP4 TF) was downregulated in A1 (Supplementary Table S3). We assume that the expression of TCP4 TF was elevated in A1 compared to A0 and may contribute to the loranthus survival under cold stress.

## 5. Conclusions

In summary, we showed evidence that loranthus seeds are sensitive to cold stress and 0°C could be an idea temperature to study the molecular changes in the seeds under cold stress. Small RNA sequencing identified 600 miRNAs including miRNA^∗^ sequences for loranthus, and 103 miRNAs were differentially expressed during the cold treatment. Some miRNAs were identified to respond to cold at early time, such as miR390a, miR160b, miR171b, and miR167c, while miR408 was identified to be functional during the cold tolerance all the time. Target prediction showed that some known cold-responsive genes could be regulated by the DEmiRs in loranthus seeds, such as ICE1, UBC, and multiple transcription factors (e.g., WKRY and TCP). Functional analysis revealed that 4, 3, and 4 DEmiRs were involved in the cold response, abiotic stimulus, and seed development/germination processes, respectively. Further, qRT-PCR was employed to validate the expression of miRNAs and target genes in the loranthus seeds during cold treatment. This is the first time to study miRNAs in loranthus, and our results may be a valuable resource for future studies of loranthus. The output of this study will improve our understanding towards the miRNA regulation of cold response in plants and benefit the loranthus breeding program.

## Figures and Tables

**Table 1:** Stimulated and suppressed miRNAs in the loranthus seeds after cold treatment for 12 h and 36 h.

miRNA	A1 vs. A0			A2 vs. A0			A2 vs. A1		
	log2FC	FDR	Regulation	log2FC	FDR	Regulation	log2FC	FDR	Regulation
miR1886	1.183	0.013	Up	1.041	0.039	Up	-0.156	0.831	NC
c55345_g4_i1_29371-3p	1.242	0.018	Up	1.010	0.081	Up	-0.237	0.764	NC
miR5067	1.062	0.032	Up	1.284	0.010	Up	0.262	0.710	NC
miR477	1.966	0.001	Up	1.552	0.026	Up	-0.373	0.646	NC
miR398_2	2.115	0.000	Up	3.057	0.000	Up	0.991	0.054	NC
miR398	1.439	0.002	Up	2.203	0.000	Up	0.815	0.097	NC
c52663_g1_i2_17575-3p	1.141	0.014	Up	2.087	0.000	Up	0.837	0.087	NC
miR5179	1.792	0.001	Up	1.717	0.003	Up	-0.014	1.000	NC
miR408	1.681	0.001	Up	3.507	0.000	Up	1.890	0.000	UP
c52663_g1_i7_17584-3p	1.232	0.013	Up	1.737	0.000	Up	0.443	0.455	NC
ath-miR779.2-3p	-1.202	0.013	Down	-1.902	0.000	Down	-0.625	0.263	NC
ath-miR2934b-5p	-5.742	0.001	Down	-5.738	0.001	Down	0.000	1.000	NC
miR156r	-1.064	0.030	Down	-1.286	0.009	Down	0.030	1.000	NC
ath-miR859-3p	-6.434	0.000	Down	-2.375	0.006	Down	4.134	0.113	NC
miR2865	-6.318	0.000	Down	-6.324	0.000	Down	0.000	1.000	NC

**Table 2:** Number of targets for differentially expressed miRNAs.

Type	Type	Number of miRNAs	Number of targets
Loranthus	ALL	479	5,610
A1 vs. A0	Total	53	501
	Upregulated	24	316
	Downregulated	29	185
A2 vs. A0	Total	36	216
	Upregulated	24	180
	Downregulated	12	39
A2 vs. A1	Total	55	624
	Upregulated	37	331
	Downregulated	19	293

**Table 3:** Key miRNAs and their targets involved in cold response and seed germination.

Function	Target	Annotation	miRNA	A1_vs._A0	A2_vs._A0	A2_vs._A1
Response to cold	c43520_g1_i1	Soluble inorganic pyrophosphatase 1, chloroplastic-like	miR4228	NC	NC	Up
	c48194_g1_i4	Phospholipase D p1-like	miR8036	Down	NC	Up
	c47711_g1_i2	Thylakoid lumenal 15 kDa protein 1, chloroplastic	aly-miR390a-3p	Up	NC	NC
	c47711_g1_i4	Hypothetical protein GLYMA_11G222100				
	c47711_g1_i3	Hypothetical protein VITISV_013914				
	c47711_g1_i1	Hypothetical protein GLYMA_11G222100				
	c51250_g1_i1	Gibberellin receptor GID1B	zma-miR164d-5p	NC	NC	Down
Response to abiotic stimulus	c29605_g1_i1	Uncharacterized protein LOC100247992	gma-miR1520o-3p	Up	NC	Down
	c34743_g1_i3	RNA pseudouridine synthase 1-like	c53051_g1_i1_18088-5p	NC	Down	Down
	c44975_g1_i2	Uncharacterized protein LOC105644596	sly-miR10539-3p	Up	NC	Down
Embryo development ending in seed dormancy	c46765_g2_i1	ruBisCO large subunit-binding protein subunit alpha	th-miR3434-5p	NC	Up	Up
	c52633_g2_i3	ATP-dependent zinc metalloprotease FtsH	MIR5998	NC	NC	Up
Seed germination	c51250_g1_i1	Gibberellin receptor GID1B	zma-miR164d-5p	NC	NC	Down
Seed dormancy process						
Mucilage extrusion from seed coat	c49078_g1_i1	Subtilisin-like protease SBT1.7	gma-miR1520o-3p	Up	NC	Down
Mucilage metabolic process involved in seed coat development						
Seed coat development	c50468_g1_i1	Subtilisin-like protease SBT1.7				

## Data Availability

The raw sequencing data can be accessed from the NCBI Sequence Read Archive (SRA) platform (http://trace.ncbi.nlm.nih.gov/Traces/sra/) under the accession number PRJNA685258.

## Supplementary Materials

Supplementary 1 Supplementary Table S1: miRNAs identified in the loranthus seeds by small RNA sequencing.

Supplementary 2 Supplementary Table S2: miRNA expression profiles of the loranthus seeds under cold stress.

Supplementary 3 Supplementary Table S3: differentially expressed miRNAs in the loranthus seeds under cold stress.

Supplementary 4 Supplementary Table S4: target prediction for differential expressed miRNAs in the loranthus seeds under cold stress.
